# Incidence and prevalence of vitiligo by gender and age in the Colombian population

**DOI:** 10.7705/biomedica.7522

**Published:** 2025-09-22

**Authors:** Paola Andrea Rueda, Sara Orozco, Juan Raúl Castro, Ángela María Londoño, Elsa María Vásquez, Andrea Arango, Carlos Esteban Builes

**Affiliations:** 1 Facultad de Ciencias de la Salud, Universidad CES, Medellín, Colombia Universidad CES Facultad de Ciencias de la Salud Universidad CES Medellín Colombia; 2 Departamento de Dermatología, Universidad CES, Medellín, Colombia Universidad CES Departamento de Dermatología Universidad CES Medellín Colombia; 3 Departamento de Dermatología, Hospital Universitario Nacional de Colombia, Bogotá, D. C., Colombia Departamento de Dermatología Hospital Universitario Nacional de Colombia Bogotá D. C Colombia; 4 Departamento de Medicina Interna, Universidad de Antioquia, Medellín, Colombia Universidad de Antioquia Departamento de Medicina Interna Universidad de Antioquia Medellín Colombia; 5 Departamento de Endocrinología, Hospital Pablo Tobón Uribe, Medellín, Colombia Departamento de Endocrinología Hospital Pablo Tobón Uribe Medellín Colombia

**Keywords:** Vitiligo, epidemiology, prevalence, incidence, Colombia, vitiligo, epidemiología, prevalencia, incidencia, Colombia

## Abstract

**Introduction.:**

Vitiligo is an acquired depigmenting skin disorder characterized by achromic macules resulting from the selective destruction of melanocytes. Epidemiological information regarding this condition remains limited in Colombia and Latin America, with prevalence varying across geographical regions.

**Objective.:**

To estimate the incidence and prevalence of vitiligo in Colombia by age, gender, and region.

**Materials and methods.:**

A retrospective study was conducted utilizing real-world clinical practice data retrieved from the national registry SISPRO *(Sistema de información para la Protección Social)* spanning the period from 2015 to 2022.

**Results.:**

Over the eight-year follow-up period (2015-2022) in Colombia, 131,494 patients were diagnosed with vitiligo. In 2016, the lowest incidence was observed, with 26 cases per 100,000 inhabitants, whereas in 2019, the highest incidence was recorded at 47 cases per 100,000 inhabitants, representing a difference of 11,175 cases between the two years. Incidence decreased in 2020 to 28.15 cases per 100,000 inhabitants. Prevalence exhibited a linear increase over the years, with the highest rates observed in 2018 and 2019.

**Conclusions.:**

This study represents the first comprehensive report on epidemiological data concerning individuals with vitiligo in Colombia, and the second in South America. It contributes to the understanding of this condition, revealing an upward trend in incidence and prevalence. These findings also provide insights into primary treatment modalities, which are probably associated with improved case registration by healthcare providers.

## Resumen

**Introducción.:**

El vitiligo es un trastorno cutáneo adquirido, caracterizado por la despigmentación de la piel y la presencia de máculas acrómicas secundarias a la destrucción selectiva de los melanocitos. En Colombia y, en general en Latinoamérica, la información epidemiológica sobre esta enfermedad es limitada y su prevalencia varía según la región geográfica.

**Objetivo.:**

Estimar la incidencia y la prevalencia del vitiligo diagnosticado en Colombia, según edad, sexo y región.

**Materiales y métodos.:**

Se llevó a cabo un estudio retrospectivo en el cual se utilizaron datos de la práctica clínica real, obtenidos del registro nacional SISPRO (sistema de información para la protección social) en el periodo 2015 -2022.

**Resultados.:**

Durante el periodo de ocho años de seguimiento se diagnosticaron 131.494 pacientes con vitiligo en Colombia. La incidencia más baja se registró en el 2016 con 26 casos por 100.000 habitantes, mientras que el valor más alto se presentó en el 2019, con 47 casos por 100.000 habitantes, lo que representa una diferencia de 11.175 casos entre los dos años. En el 2020, hubo una reducción con 28,15 casos por 100.000 habitantes. La prevalencia tuvo un incremento lineal a lo largo del período, con tasas más elevadas en el 2018 y el 2019.

**Conclusiones.:**

Este es el primer estudio en el que se reporta información epidemiológica sobre vitiligo en Colombia y el segundo en Sudamérica, lo que lo convierte en un aporte valioso para ampliar el conocimiento sobre la enfermedad. Se resalta el aumento tanto de la incidencia como de la prevalencia de vitiligo, así como en los procedimientos empleados como tratamiento de primera línea, quizá asociado al mayor registro por parte del personal sanitario. Este trabajo sienta las bases para futuras investigaciones sobre la enfermedad.

Vitiligo is an acquired depigmenting skin disorder characterized by achromic macules resulting from the selective destruction of melanocytes. The exact pathogenesis remains elusive and has been linked to various mechanisms, including metabolic abnormalities, oxidative stress, inflammatory mediators, and autoimmune responses. Although it can manifest at any age, onset is typically observed during the second to third decade of life, with a slightly higher prevalence among women than among men ^1^.

The prevalence of vitiligo varies across geographical regions, with data availability depending on the location. Globally, the prevalence of the disease is reported as 0.1% in Asia, 0.4% in Africa, 0.2% in America, 0.4% in Europe, and 1.2% in Oceania ^1^^-^^3^. Stratification by publication year reveals a prevalence of 0.6% before the 1980s, which decreased to 0.2-0.3% in the 1980s, and then increased to 0.6% in the first half of the 1990s. Prevalence rates by age group were reported as 0.2% for individuals aged 0 to 19 years, 0.4% for those aged 20 to 39 years, and 0.7% for subjects aged 60 or older, indicating a positive correlation with age ^1^^,^^2^^,^^4^.

Prevalence estimates of vitiligo vary significantly across populations due to differences in study design and data collection methods, including patient self-reports, medical records, and claims ^5^^,^^6^.

One of the most extensive epidemiological studies of vitiligo in the United States reported a prevalence of 0.2% and an annual incidence of 22.6 patients per 100,000 individuals. This study identified approximately 528,000 prevalent cases and 74,600 new cases per year. Incidence and prevalence were higher among older individuals; the latter rate was nearly double in adults compared to pediatric patients. Furthermore, no significant differences in incidence or prevalence were found between men and women ^2^.

As noted, the prevalence of vitiligo varies by age, gender, and ethnicity. Although the disease is encountered worldwide, vitiligo appears to be more prevalent in Asian and African populations, whereas data from South American countries, including Colombia, remains scarce ^7^.

The lack of comprehensive information regarding the epidemiology of vitiligo in Latin America, specifically in Colombia, underscores the need for detailed studies. This retrospective analysis aims to elucidate the epidemiological profile of vitiligo in Colombia from 2015 to 2022, focusing on factors such as gender, age, and region. Based on reports from a comprehensive database that collects information from all providers within the public health system, this study seeks to provide valuable insights into the prevalence and distribution of vitiligo in the Colombian population.

## Materials and methods

### Study design and population

A retrospective ecological study was conducted using data from the national database SISPRO *(Sistema de Información para la Protección Social),* which systematically compiles information from Colombia's social security healthcare system. Reporting to SISPRO is mandatory for healthcare personnel during every outpatient or hospital visit, ensuring standardized recording of patient information. Data collection spanned from 2015 to 2022 and included patients of all ages, genders, and regions of Colombia with at least one record coded under the International Classification of Diseases, 10^th^ Revision (ICD-10) code for vitiligo: L80.

### Variables

Sociodemographic variables (age and gender) and healthcare resources use (consultations and procedures) for patients with vitiligo were included in the analysis. The population counts and distributions by region, age, and sex -used to estimate incidence and prevalence- were obtained from the affiliates database of the general social security health system and from the DANE *(Departamento Administrativo Nacional de Estadística),* based on the latest national census conducted in 2018.

Prevalence of vitiligo was calculated as the number of vitiligo cases for each age group, reported in the SISPRO, divided by the corresponding population counts reported for each region by the DANE. Incidence was estimated similarly, using the number of new vitiligo cases as the numerator and the population counts from the DANE as the denominator.

### Statistical analysis

Data was analyzed using descriptive statistics. Continuous variables were summarized using central tendency (means and medians) and dispersion (standard deviation and range) measures, whereas categorical variables were expressed as absolute numbers and relative frequencies.

Crude rates were adjusted for the total population by year and age group (when needed). Age-specific rates were calculated using Epidat, version 3.1 *(Dirección Xeral de Saúde Pública, Xunta de Galicia,* PAHO-WHO). Incidence and prevalence rates were estimated per 100,000 inhabitants.

### Data availability

The datasets analyzed during the current study are available from the corresponding author upon reasonable request.

### Publication consent

Informed consent was not required as this retrospective study used information reported by the national registry SISPRO *(Sistema de Información para la Protección Social)* between 2015 and 2022.

## Results

**Figure 1:**
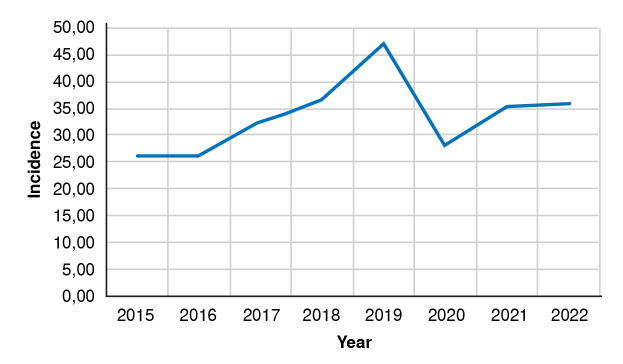
Incidence of vitiligo (rate per 100,000 inhabitants) in the Colombian population, 2015-2022

During the eight-year follow-up period (2015-2022), 131,494 patients were diagnosed with vitiligo in Colombia, of whom 83,877 were women. The lowest incidence occurred in 2016 (26 cases per 100,000 inhabitants) and the highest in 2019 (47 cases per 100,000 inhabitants), corresponding to a difference of 11,175 cases between the two years. Incidence decreased in 2020 to 28.15 cases per 100,000 inhabitants, followed by an increase in 2021 and 2022 (table 1 and figure 1).

**Table 1:** Annual incidence of vitiligo per 100,000 inhabitants in the Colombian population, 2015-2022

Year	New cases	Population estimates	Annual incidence
2015	12,104	46'313,898	26.13
2016	12,197	46'830,116	26.05
2017	15,436	47'419,200	32.55
2018	17,785	48'258,494	36.85
2019	23,279	49'395,678	47.13
2020	14,189	50'407,647	28.15
2021	17,952	50'999,108	35.20
2022	18,552	51'682,692	35.90

**Figure 2:**
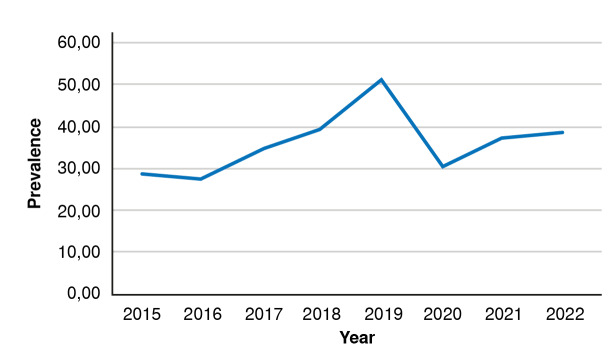
Prevalence of vitiligo in the Colombian population adjusted for the total population for each year

Prevalence exhibited a linear increase over the years, with higher rates in 2018 and 2019 compared to 2015 and 2016, followed by a marked deceleration in 2020 (figure 2). The highest number of vitiligo patients was reported in 2019, equivalent to a rate of 50.9 cases per 100,000 inhabitants. Table 2 displays the prevalence rates per year.

**Table 2:** Prevalence of vitiligo per 100,000 inhabitants in the Colombian population, 2015-2022

Year	Total cases	Population estimates	Prevalence
2015	13,211	46'313,898	28.52
2016	12,955	46'830,116	27.66
2017	16,313	47'419,200	34.40
2018	19,206	48'258,494	39.80
2019	25,156	49'395,678	50.93
2020	15,439	50'407,647	30.63
2021	19,093	50'999,108	37.44
2022	19,951	51'682,692	38.60

**Figure 3:**
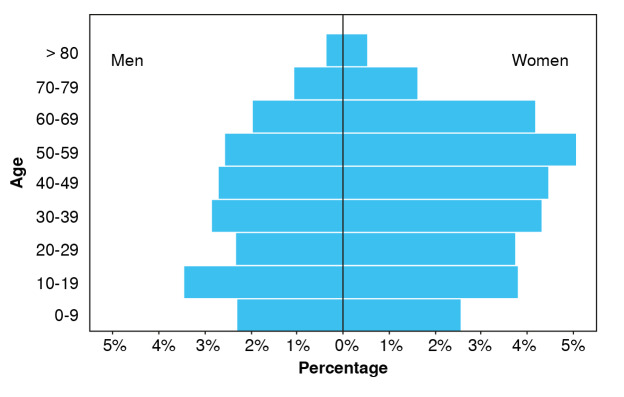
Prevalence of vitiligo (rate per 100,000 inhabitants) in the Colombian population by sex and age group

In the analysis by age group, two peaks of incidence were observed: the highest occurred in the second decade of life (10-19 years), consistent with previous literature reports, and the second peak was identified in the sixth decade of life (50-59 years) among women and the fourth decade of life (3039 years) among in men (table 3 and figure 3).

**Table 3:** Prevalence of vitiligo per 100,000 inhabitants by sex and age group, 2015-2022

Age	Male	Female
0-9	15.75	17,73
10-19	21.92	23.96
20-29	15.71	22.57
30-39	18.08	24.42
40-49	16.36	25.04
50-59	15.03	28.38
60-69	10.22	19.20
70-79	5.13	7.56
>80	1.54	2.01

**Table 4:** New and total cases of vitiligo, patient visits, and procedures performed, 2015-2022

Year	New cases	Total cases	Office visits	Procedures
2015	12,104	13,211	28,200	2,877
2016	12,197	12,955	20,558	2,715
2017	15,436	16,313	26,019	3,231
2018	17,785	19,206	33,712	3,967
2019	23,279	25,156	40,689	5,213
2020	14,189	15,439	26,446	3,348
2021	17,952	19,093	31,823	4,060
2022	18,552	19,951	39,580	3,919

This study assessed the frequency of visits to primary care physicians and specialists (including dermatologists) by vitiligo patients. Table 4 displays the number of patients attending consultations per year, including initial and follow-up visits. The number of consulting patients increased in 2019 and 2022, while it diminished in 2016 and 2017. This trend differs from the incidence and prevalence rates of the disease, which shows that the most significant change occurred in 2020. The age-adjusted incidence rate of vitiligo for 2022 was 40.38 per 100,000 inhabitants (95% CI: 39.82-40.94).

**Figure 4:**
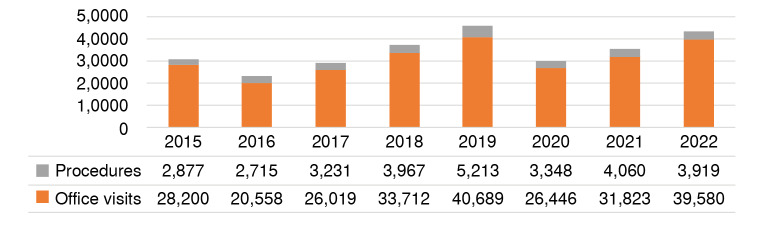
Number of patients diagnosed with vitiligo attending initial consultations and follow-up visits, and procedures performed.

Procedures related to vitiligo, such as phototherapy, microdermabrasion, and micrografting, increased in 2019. However, unlike consultations, no marked deceleration was observed in 2020. Conversely, the year with the fewest procedures performed was 2016 (figure 4).

**Figure 5:**
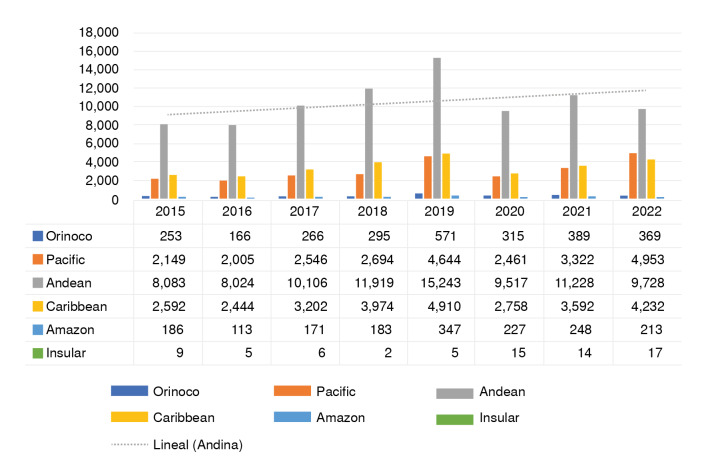
Vitiligo total of cases in Colombia by regions, 2015-2022.

Regarding regional incidence, the Andean region reported the highest rates of vitiligo over the eight years, with the department of Antioquia registering the largest number of cases. The Caribbean region ranked second (figure 5).

## Discussion

This study represents the first one aimed to estimate the epidemiology of vitiligo in Colombia, through a retrospective analysis based on data from the national registry SISPRO.

Vitiligo is a chronic skin condition characterized by a complex pathophysiology that, despite numerous advancements, remains elusive ^8^. For years, it has been considered a disease with primarily aesthetic consequences; however, this is far from the case ^9^. Vitiligo is the result of autoimmunity, like type 1 diabetes, psoriasis, multiple sclerosis, and inflammatory bowel disease. It is associated with different autoimmune diseases; for example, approximately 15% to 25% of patients have thyroid disease ^10^. This disorder profoundly affects patients' quality of life. Many cases emerge before the age of 20, subjecting individuals to stigmatization during a crucial period of personal, professional, and psychological development ^10^.

The economic impact of vitiligo is considerable, with treatment expenses including high direct and indirect costs, such as prescribed medications, medical consultations, phototherapy, lost workdays, social avoidance, and diminished quality of life. These factors strain patients' financial resources, time, and energy.

Recognizing that vitiligo is more than a mere cosmetic concern is crucial. One of the most pressing challenges is to understand that this condition requires effective - already existing- treatments for its management ^11^. However, the availability of vitiligo treatments is limited; so far, those available often fail to induce consistent repigmentation. Moreover, discontinuation of most treatments results in relapse in nearly 40% of patients within the first year, leading to resignation and a reluctance to seek medical attention due to the uncertain prognosis ^9^^,^^12^.

Epidemiological findings of this study revealed a female predominance and an increase in vitiligo prevalence in Colombia between 2018 and 2019, followed by a gradual decline in 2020 and 2021. This decline coincided with the onset of COVID-19 in March 2020 and was likely influenced by reduced patient attendance at healthcare services, affecting case reporting.

This study estimated an incidence of 0.27% and a prevalence of 0.29%, consistent with a published meta-analysis about vitiligo prevalence worldwide, which indicated an incidence of 0.2% for the Americas ^1^. The highest incidence rates were identified among children and adolescents (ages 1019), a finding aligned with the global epidemiology of vitiligo. Nonetheless, a secondary peak was observed, with gender-specific differences: in men during their fourth decade and in women during their sixth. Age-stratified prevalence reveals the highest rates among individuals aged 50-60, demonstrating a gradual rise in vitiligo prevalence with age ^1^.

When examining vitiligo epidemiology across regions of Colombia, the Andean region had the highest number of reported cases, particularly the department of Antioquia, which surpassed Bogotá, the country's largest city. Conversely, the insular region had the lowest number of reported cases due to its smaller population.

Analysis of healthcare resource utilization showed a linear increase in procedures, indicating a rising trend in the use of phototherapy, a frontline treatment for vitiligo. This escalation suggests a growing integration of phototherapy into routine clinical practice.

The observed increase in the incidence and prevalence of vitiligo in Colombia may be explained by various factors. Enhanced case reporting owing to mandatory notification and awareness campaigns, coupled with the introduction of new targeted therapies, likely improved detection. Furthermore, initiatives promoting inclusivity by commercial entities may have helped mitigate the stigma surrounding the condition, potentially encouraging more vitiligo patients to seek treatment.

However, it is important to consider some limitations of this study. the database used is a national registry, it may contain coding constraints, data collection errors, and incomplete or inconsistent information. Nevertheless, such databases represent a valuable source of information for epidemiological studies due to the large amount of compiled data and the built-in quality controls.

This study offers vital epidemiological insights into vitiligo in Colombia, representing the first national report and the second in South America. These findings contribute to increasing awareness of the disease burden among the general population and the healthcare actors. Furthermore, they lay the groundwork for future research, which may utilize additional methodologies to calculate the national prevalence of vitiligo more accurately.
